# Long-Term Effect of a Vaccine Targeting Endothelin-1 Receptor Type A in Pulmonary Arterial Hypertension

**DOI:** 10.3389/fcvm.2021.683436

**Published:** 2021-06-17

**Authors:** Yong Dai, Zhihua Qiu, Wenrui Ma, Chang Li, Xiao Chen, Xiaoxiao Song, Zeyang Bai, Dingyang Shi, Jiayu Zheng, Guangwei Pan, Yuhua Liao, Mengyang Liao, Zihua Zhou

**Affiliations:** ^1^Department of Cardiology, Union Hospital, Tongji Medical College, Huazhong University of Science and Technology, Wuhan, China; ^2^Institute of Cardiology, Union Hospital, Tongji Medical College, Huazhong University of Science and Technology, Wuhan, China; ^3^Key Lab of Molecular Biological Targeted Therapies of the Ministry of Education, Union Hospital, Tongji Medical College, Huazhong University of Science and Technology, Wuhan, China

**Keywords:** pulmonary arterial hypertension, endothelin-A receptor, vaccine therapy, durable efficacy, vascular remodeling

## Abstract

**Background:** Previously, we invented a therapeutic vaccine targeting the endothelin-A receptor (termed ETRQβ-002). ETRQβ-002 successfully prevented the remodeling of pulmonary arterioles (PAs) and right ventricle (RV) without significant immune-mediated damage in experimental pulmonary arterial hypertension (PAH) mice models.

**Objective:** Here, we aim to further evaluate the long-term effects of ETRQβ-002.

**Methods:** PAH mice model was induced by a combination of subcutaneous injection with Sugen5416 and chronic hypoxic conditions (10% O_2_). PAH mice were immunized with ETRQβ-002 at different time points, and the experiment lasted for 21 weeks. Hemodynamic, histological, and biochemical analyses were conducted to evaluate the long-term effects of ETRQβ-002.

**Results:** We demonstrated that the titer of the specific antibody against ETR-002 could be maintained chronically after periodic booster immunization in PAH mice. Long-term reduction of right ventricular systolic pressure and amelioration of PA remodeling by ETRQβ-002 were confirmed. Moreover, we found that ETRQβ-002 also exerted antiproliferation, anti-inflammation, and antifibrosis effects in PA remodeling. Besides, ETRQβ-002 durably limited pathological RV hypertrophy and fibrosis. Finally, no immune-mediated damage was observed in hepatic or renal function or by pathology in liver and kidney during the long-term administration of ETRQβ-002.

**Conclusion:** Our findings indicate that ETRQβ-002 provides long-term therapeutic effects in Sugen/hypoxia-induced PAH animals and offers a promising clinical prospect for PAH treatment.

## Introduction

Pulmonary arterial hypertension (PAH) is a chronic life-threatening disease characterized by pulmonary arteriole (PA) remodeling. Pathological changes of PA in PAH, including thickening of intimal and medial layers, fibrotic vasculopathy, and perivascular inflammatory cell infiltration (1–3), lead to a progressive elevation of pulmonary arterial pressure and, eventually, right ventricular failure. In recent years, clinical, functional, and hemodynamic improvement in PAH patients has been achieved with the application of a collection of targeted drugs such as endothelin-receptor antagonists, phosphodiesterase type 5 inhibitors, and prostacyclin derivatives (4). Despite the great advances in pharmacological therapy, the quality of life and prognosis of PAH patients remain poor, with a 5-year survival rate of 69% (5). Thus, it is urgent to explore novel and affordable therapeutic approaches for the treatment of PAH.

Therapeutic vaccine is a new method for treating chronic diseases (6). Unlike preventive vaccines, therapeutic vaccines mainly aim at self-antigens. Self-antigen alone does not always break tolerance and lead to an immune response. To improve the immunogenicity, the self-antigens are conjugated with foreign carriers, such as virus-like particles (VLPs). Through a series of immune responses, self-antigen–carrier conjugate would break through self-immune tolerance and induces a specific antibody, which can bind to the antigen, thereby exerting a therapeutic effect. Meanwhile, the self-epitopes are segregated from Tc (cytotoxic T cell) due to the structure of the peptide-based vaccine, and there are no self–T-cell epitope-specific T-cell clones to cause autoimmune damage (7). Therefore, vaccines comprising self-antigens coupled to carriers present candidates capable of achieving efficacious antibody levels while fulfilling the necessary safety criteria.

It is now well-known that the abnormal activation of endothelin (ET) system, especially ET-1 and ET(A) receptors, plays a critical role in the pathogenesis of PAH (8). A series of clinical studies have shown that ET receptor antagonists effectively improve the symptoms and clinical deterioration time of patients with PAH (9). Our previous study has demonstrated that a therapeutic vaccine targeting ETAR (termed ETRQβ-002) effectively decreases right ventricular systolic pressure (RVSP) in experimental PAH rodent animals. In addition, ETRQβ-002 vaccine alleviates pathological remodeling of PAs and right ventricular, while exhibiting a satisfactory safety profile (10). However, in the previous research, the period of observation was relatively short in PAH animals, rendering it difficult to evaluate the durable efficacy of ETRQβ-002. Moreover, prophylactic administration of ETRQβ-002 markedly improved hemodynamics, right ventricle (RV) hypertrophy, and PA remodeling. However, whether therapeutic effect could be provided when ETRQβ-002 is administered after the establishment of hemodynamic PH also needs to be investigated.

In this study, a relatively long-lasting experiment was performed in Sugen/hypoxia (SuHx)–induced PAH mice. During the process of PAH induction, mice were immunized with ETRQβ-002 at different points of time, aiming to verify if ETRQβ-002 is capable of exerting long-acting protective effects in well-established PAH mice. Moreover, mounting evidence shows that ET system is implicated in various pathological processes including proliferation (11), inflammation, and fibrosis (12, 13), all of which are vital initiators of PA remodeling in PAH. The main signal pathways involved in these pathological processes in PAH including mitogen-activated protein kinases (MAPKs) and transforming growth factor β (TGF-β) pathways. Nuclear factor-κB (NF-κB), which is a transcription factor and plays a critical role in inflammation regulation, is also involved in PAH pathogenesis (14, 15). Based on these facts, we proposed and verified the hypothesis that ETRQβ-002 may offer potential antiremodeling effects in PA partly through attenuating the above pathological processes.

## Materials and Methods

### Ethics Statement

The study was carried out in strict accordance with the Guidelines for the Care and Use of Laboratory Animals (Science & Technology Department of Hubei Province, PR China, 2005). The protocol was approved by the Ethics Committee of Tongji Medical College of Huazhong University of Science and Technology, China. Mice were housed in a specific pathogen-free laboratory at 22°C, with a 12-h light–dark cycle, and provided with sterile water, standard chow diet.

### Vaccine Preparation and Experimental PH Model

ETRQβ-002 vaccine was prepared as previously described (10). Male C57BL/6 mice (Vital River, Beijing, China) aged 6 weeks were randomly divided into five groups: (1) a control group received only vehicle (Con, *n* = 14); (2) a hypoxia- and Su5416-exposed group (SuHx, *n* = 26); (3) a SuHx + ETRQβ-002(s) group (simultaneous vaccination and SuHx exposure) (*n* = 26); (4) a SuHx + ETRQβ-002(e) group (vaccination after established PAH) (*n* = 26); (5) a SuHx + VLP group (*n* = 26). All mice except the control group experienced a weekly injection of Su5416 (20 mg/kg, TargetMol) and were exposed to chronic normobaric hypoxia in a hypoxic chamber flushed with a mixture of N_2_ and room air (10% O_2_). The mice in the SuHx + ETRQβ-002(s) group were immunized with ETRQβ-002 vaccine (100 μg per mice) on days 0, 14, 28, 42, and 77 before exposure to chronic hypoxia, whereas the mice in the SuHx + ETRQβ-002(e) group were immunized with ETRQβ-002 vaccine (100 μg per mice) on days 28, 42, 56, 70, and 105 when the hypoxia-induced PAH has been established. After being exposed to chronic normobaric hypoxia for 3 weeks, the animals were followed by re-exposure to normoxia (21% O_2_) for 18 additional weeks. ETRQβ-002 vaccine–specific peptide antibody titer in groups 3 and 4 was detected by enzyme-linked immunosorbent assay (ELISA) every 2 weeks after the second immunization. All mice were sacrificed on day 148.

### Hemodynamic Measurements

Mice were anesthetized with ketamine (100 mg/kg). RVSP was measured as previously reported (16) with polyethylene catheters (Scientific Commodities Inc., Lake Havasu City, Arizona) *via* a polygraph system (LabChart 7.3.7; AD Instruments, New South Wales, Australia) using pressure catheters (SPR-671; Millar, Texas, USA).

### Morphological Assessment

Lungs and RV specimens were washed by phosphate-buffered saline and immediately fixed in 10% formalin and embedded in paraffin as previously described (16). Cardiomyocyte diameters of the RV were determined through wheat germ agglutinins (WGAs). The fibrotic area (%) of PA and RV was evaluated through Masson trichrome staining. PA remodeling was calculated by hematoxylin-eosin and α-smooth muscle actin (anti–α-SMA; Sigma). The percentage of medial wall thickness (MT%) of vessels (20- to 70-mm diameter) was measured, and the degree of muscularization of vessels was categorized as non-muscular, partially muscular or fully muscular, according to the previously described (10). Parts of fresh renal cortex were immediately fixed in 0.25% glutaraldehyde for transmission electron microscopy (TEM). All of the analyses were performed blinded to the study conditions.

### Lung Immunohistochemistry and Immunofluorescence

Lung sections was performed with immunohistochemical staining (including PCNA and CD68) and immunofluorescence staining (including α-SMA, Ki67, CD45, and collagen III) according to the previous method (17). The number of proliferating marker cells and inflammatory cells in vessels and perivascular tissues was counted in a blinded fashion in 15-25 randomly chosen high-powered fields. The α-SMA–collagen III colocalization was expressed as percent double-positive area/sum total area of stain for each protein of PAs.

### Protein and mRNA Analysis

RNA isolation, cDNA synthesis, quantitative real-time polymerase chain reaction (qRT-PCR), protein extraction, and Western blotting were performed as previously described (17). qRT-PCR on resulting cDNA were performed to determine relative expression of each gene of interest, determined by the 2^−ΔΔCq^ method and normalized to the relative expression of 18S. Sequences of primers are provided in Supplementary Table 1. Densitometric analyses of Western blot results were quantified using Image Lab software (Bio-Rad Laboratories, CA, USA) and were expressed relative to the mean of the control group. All the antibodies used in Western blot are listed in Supplementary Table 2.

### Statistical Analysis

Data were initially screened for normality using the Shapiro–Wilk test and were expressed as mean ± standard error of the mean (SEM). A one-way analysis of variance (ANOVA) using Bonferroni's method (for comparison of more than two groups) was used for the statistical analyses. The differences in some observations [MT, cross-sectional area (CSA), PCNA, Ki67, CD45, CD68, fibrosis, and colocalization of Pas] were analyzed using a mixed-effects model. If the assumption of the homogeneity of variances was violated, as assessed with the Levene test for equality of variances, Welch ANOVA was used with the Games–Howell *post hoc* test for pairwise comparisons. The calculation was carried out using the statistical program SPSS version 19.0. *P* < 0.05 was accepted as significant.

## Results

### ETRQβ-002 Exerted Long-Term Effects of Attenuating PAH and Reducing PA Thickening in SuHx-Induced PAH

**Figure 1:**
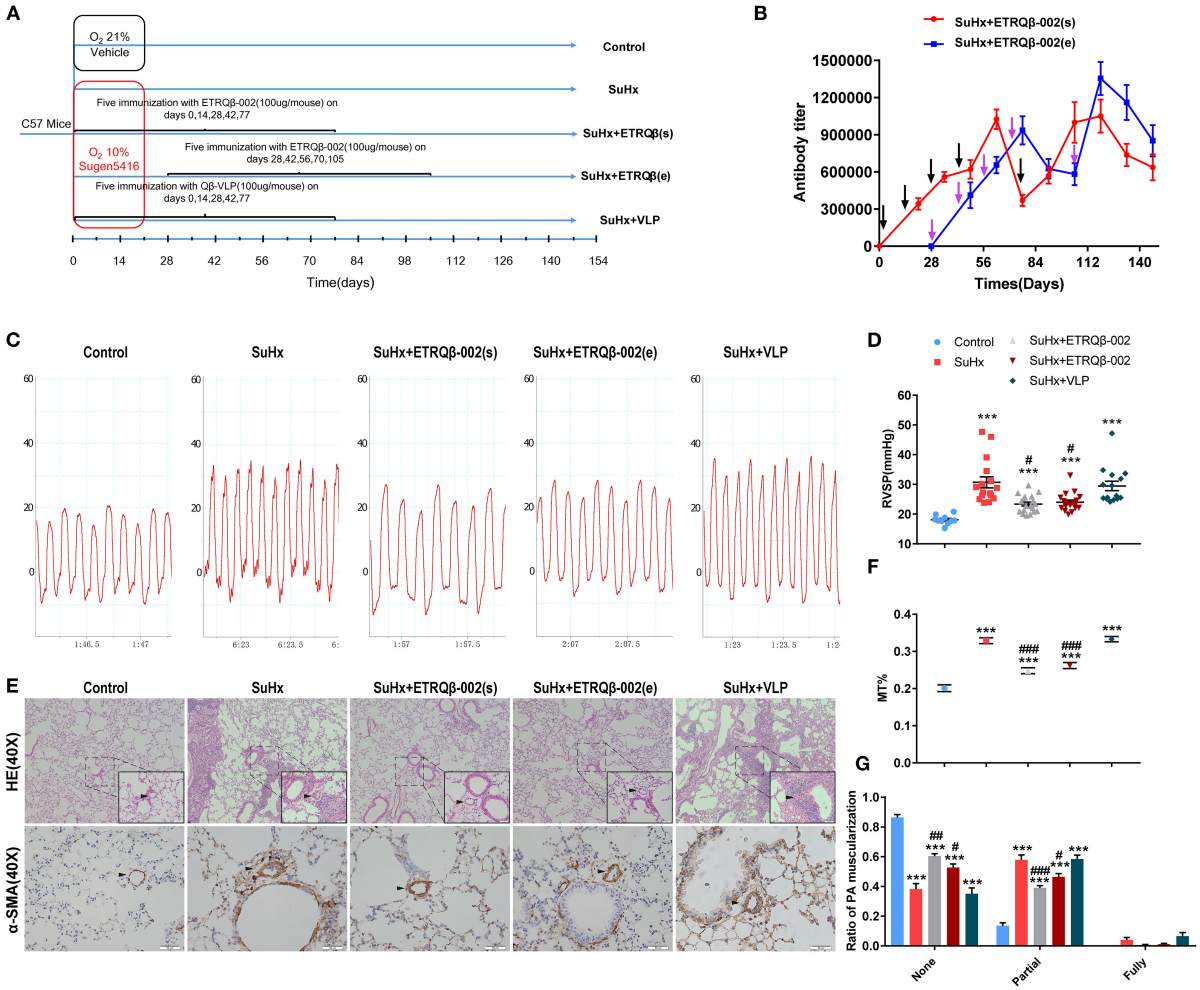
Long-term efficacy of ETRQβ-002 vaccine in SuHx-induced PAH mice. **(A)** The progress map of SuHx-induced mice PAH model. **(B)** The ETRQβ-002 specific antibody titers were screened on days 21, 35, 49, 63, 77, 91, 105, 119, 133, and 147 in the SuHx + ETRQβ-002(s) group (*n* = 10), whereas on days 49, 63, 77, 91, 105, 119, 133, and 147 in the SuHx + ETRQβ-002(e) group (*n* = 10). The black and purple arrows refer to the ETRQβ-002 injections in the SuHx + ETRQβ-002(s) group and SuHx + ETRQβ-002(e) group, respectively. **(C)** Representative images of the pulmonary hypertension in the five groups. **(D)** RVSP measured by right cardiac catheterization on day 148 (*n* = 10–19). **(E)** Representative photographs of hematoxylin-eosin (HE) and α-smooth muscle actin (α-SMA) staining of lung sections from each group (scale bar = 50 μm). **(F)** The percentage medial wall thickness (MT%) of vessels (diameter, 20–70 μm; *n* = 8–10). **(G)** Ratio of PA muscularization (none, partial, and fully; *n* = 8–10). The black arrows refer to the PAs. All data are expressed as the mean ± SEM. ^***^P < 0.001 vs. the control group; ^#^*P* < 0.05, ^##^*P* < 0.01, ^###^*P* < 0.001 vs. the SuHx group.

To identify durable efficacy of ETRQβ-002 and whether therapeutic effect could be exerted in established PAH model, we randomly assigned male C57BL/6 mice into five groups, and the study duration for this PAH model was ~150 days (Figure 1A). After four basic immunizations, special antibody in serum of mice immunized with ETRQβ-002 was detected. ELISA test revealed that antibody titers in the SuHx + ETRQβ-002(s) group and the SuHx + ETRQβ-002(e) group were 1:1,000,000 to 1:1,100,000 and 1:900,000 to 1:1,100,000, respectively. Five weeks after the fourth immunization, a booster immunization was performed because of the lowered antibody titer. Afterward, the antibody titers increased again and maintained at a high level until sacrifice (Figure 1B).

Further, the long-term protective efficacy was investigated. Hemodynamic result showed that RVSP significantly increased in all the groups treated with SuHx. However, RVSP in the SuHx + ETRQβ-002(s) and SuHx + ETRQβ-002(e) groups decreased markedly compared with the SuHx group (ETRQβ-002(s), 23.38 ± 0.67 mm Hg, *P* = 0.012; ETRQβ-002(e), 23.99 ± 0.72 mm Hg, *P* = 0.023; SuHx 30.72 ± 1.86 mm Hg; Figures 1C,D). No significant difference was detected between the SuHx + ETRQβ-002(s) group and the SuHx + ETRQβ-002(e) group. Then, PA pathological remodeling including media wall thickness (MT) and fraction of muscularized arteries were evaluated. As the results shows, the MT% remarkably increased in all the groups except the control group. However, the MT% in the SuHx + ETRQβ-002(s) group and the SuHx + ETRQβ-002(e) group was significantly lower than that of the SuHx group (ETRQβ-002(s), 0.248 ± 0.008, *P* < 0.001; ETRQβ-002(e), 0.262 ± 0.008, *P* < 0.001; SuHx 0.329 ± 0.007; Figures 1E,F). In addition, ETRQβ-002 also reversed the partial muscularization of PAs (ETRQβ-002(s), 0.390 ± 0.016, *P* < 0.001; ETRQβ-002(e), 0.464 ± 0.023, *P* = 0.015; SuHx 0.579 ± 0.032; Figure 1G). Fraction of fully muscularization increased in mice treated with SuHx was decreased by ETRQβ-002 administration, but did not reach statistical significance. These results indicated that given periodic enhancement of immunity, ETRQβ-002 provides a durable efficacy in established PAH model.

### ETRQβ-002 Ameliorated Proliferation of PA and Inhibited Phosphorylation of ERK1/2 and p38 in SuHx-Induced PAH

**Figure 2:**
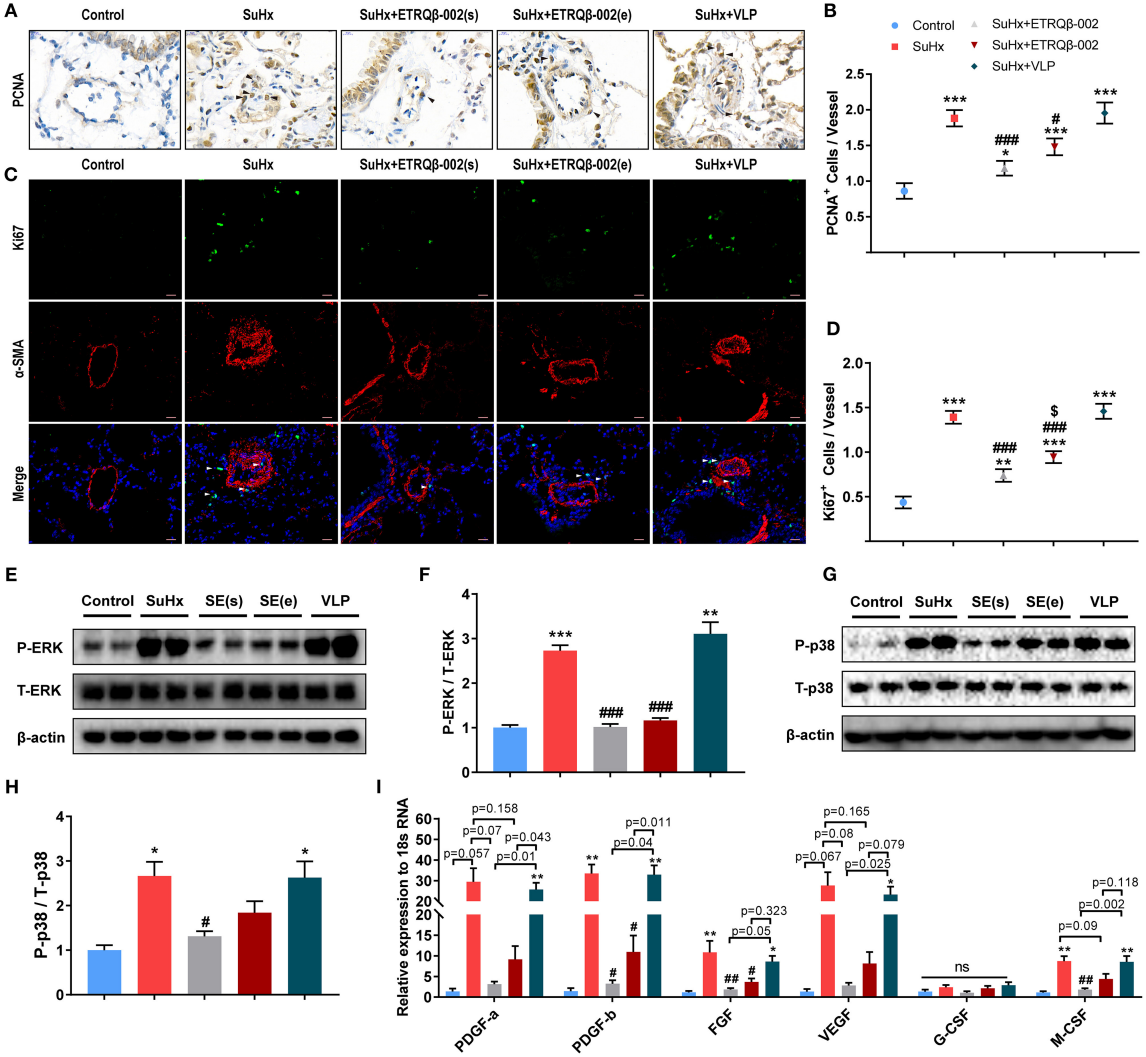
ETRQβ-002 vaccine effectively reduced proliferation response of PA in SuHx-induced PAH mice. **(A)** Representative photographs of PCNA immunohistochemistry staining of lung sections from each group (scale bar = 10 μm). **(B)** The number of PCNA-positive cells in vessels and perivascular tissues (*n* = 6). **(C)** Representative photographs of Ki67/α-SMA double-immunofluorescence staining of lung sections from each group (scale bar = 20 μm). **(D)** The number of Ki67-positive cells in vessels and perivascular tissues (*n* = 6). **(E,F)** Representative immunoblot, corresponding densitometric analysis of pERK and total ERK (*n* = 6). **(G,H)** Representative immunoblot, corresponding densitometric analysis of pp38 and total p38 (*n* = 6). **(I)** Quantitative RT-PCR (qRT-PCR) analysis of growth factors in lung homogenate from five groups (*n* = 4–5). The black and white arrows refer to the PCNA-positive cells and the Ki67-positive cells, respectively. All data are expressed as the mean ± SEM. ^*^P < 0.05, ^**^P < 0.01, ^***^P < 0.001 vs. the control group. ^#^*P* < 0.05, ^*##*^*P* < 0.01, ^*###*^*P* < 0.001 vs. the SuHx group. ^*$*^*P* < 0.05 vs. the SuHx + ETRQβ-002(s) group. ns, no significant difference.

Similar to cancer, PAH is a proliferative disease characterized by accumulation of diverse vascular cells in the pulmonary arterial wall (2, 14). To evaluate the antiproliferative efficacy of ETRQβ-002, PCNA (proliferating cell nuclear antigen) immunohistochemical staining, and Ki67/α-SMA double-immunofluorescence staining were performed in lung sections of all the mice. Results demonstrated that SuHx exposure observably increased the number of PCNA-positive and Ki67-positive cells in vessels and perivascular tissues (Figures 2A–D), but ETRQβ-002 effectively restrained this change. However, we also found a larger number of proliferating marker cells in the ETRQβ-002 (e) group than the ETRQβ-002(s) group.

Mounting evidence shows that aberrant ERK and p38 activation play a potent role in PAH pathobiology (18, 19). Consistent with the antiproliferative effect of ETRQβ-002 in our previous study *in vitro*, elevated phosphorylation of ERK1/2 observed in SuHx-exposed lungs was markedly attenuated by ETRQβ-002 administration (ETRQβ-002(s), 63% decrease, *P* < 0.001; ETRQβ-002(e), 58% decrease, *P* < 0.001; Figures 2E,F). Similar result was found in phosphorylation of p38 (ETRQβ-002(s), 51% decrease, *P* = 0.036; ETRQβ-002(e), 31% decrease, *P* = 0.332; Figures 2G,H). Moreover, compared to the SuHx group, increased mRNA expression of growth factors associated with PAH in lung was also significantly down-regulated in mice immunized with ETRQβ-002 (Figure 2I). It is worth noting that phosphorylation level of ERK1/2 and p38 and mRNA expression of growth factors in the ETRQβ-002(e) groups are slightly higher than those in the ETRQβ-002(s) groups. These results indicated that antiproliferative effects were more superior when mice were immunized with ETRQβ-002 earlier during the process of PAH induction.

### ETRQβ-002 Attenuated Inflammation of PA and Inhibited NF-κB Pathway in SuHx-Induced PAH

**Figure 3:**
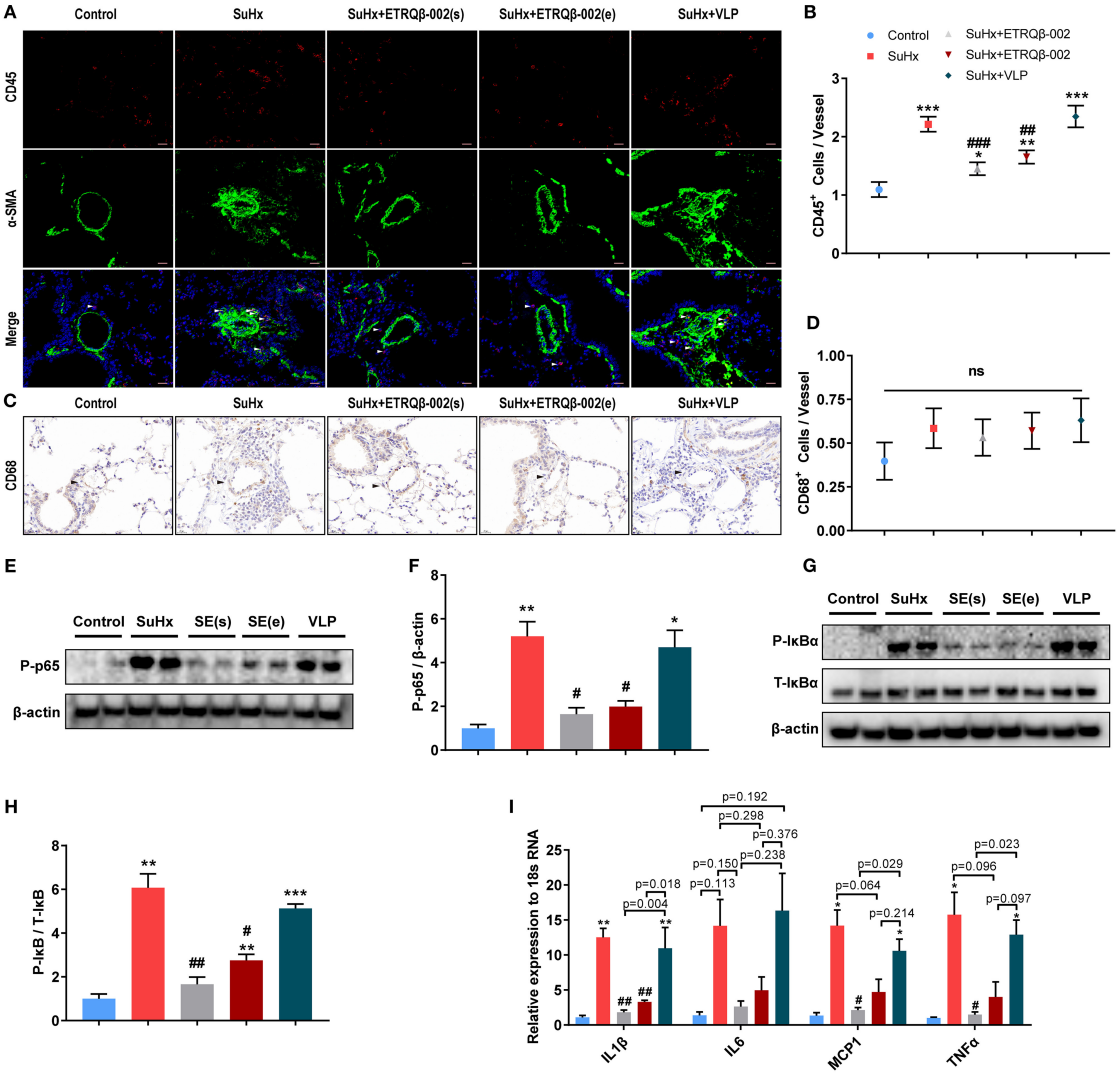
ETRQβ-002 vaccine effectively reduced inflammation response of PA in SuHx-induced PAH mice. **(A)** Representative photographs of CD45/α-SMA double-immunofluorescence staining of lung sections from each group (scale bar = 20 μm). **(B)** The number of CD45-positive cells in vessels and perivascular tissues (*n* = 5). **(C)** Representative photographs of CD68 immunohistochemistry staining of lung sections from each group (scale bar = 20 μm). **(D)** The number of CD68-positive cells in vessels and perivascular tissues (*n* = 5). **(E,F)** Representative immunoblot, corresponding densitometric analysis of pIκB and total IκB (*n* = 6). **(G,H)** Representative immunoblot, corresponding densitometric analysis of pp65 and total p65 (*n* = 6). **(I)** Quantitative RT-PCR (qRT-PCR) analysis of proinflammatory factors in lung homogenate from five groups (*n* = 4–5). The white and black arrows refer to the CD45-positive cells and the PAs, respectively. All data are expressed as the mean ± SEM. ^*^P < 0.05, ^**^P < 0.01, ^***^P < 0.001 vs. the control group. ^#^*P* < 0.05, ^*##*^*P* < 0.01, ^*###*^*P* < 0.001 vs. the SuHx group. ns, no significant difference.

In addition to the accumulation of vascular cells, PA remodeling in PAH is also characterized by exaggerated perivascular infiltration of inflammatory cells (1, 2). Then, staining and quantification of representative inflammatory and immune cells including macrophages and lymphocytes in lung were carried out. As shown in Figures 3A,B, there was a significant increase of the number of perivascular CD45^+^ cells in all SuHx–exposed lung, but the number of those was significantly lower in the ETRQβ-002(s) and ETRQβ-002(e) groups. Our data suggested that ETRQβ-002 administration decrease perivascular infiltration of lymphocytes cells in PAH. However, no significant difference was observed in number of perivascular CD68^+^ cells in all groups (Figures 3C,D).

It is universally acknowledged that, as a leading regulator of inflammation, NF-κB plays a central role in the inflammatory response of PAH. Activated NF-κB pathway promotes expression of various genes encoding proinflammatory cytokines and therefore causes vascular inflammation (15, 20, 21). To determine whether ETRQβ-002 could inhibit activation of NF-κB, the protein expression of phosphor-NF-κB (p-p65), which indicates NF-κB activity, was assessed in lung tissue homogenate. The results demonstrated that ETRQβ-002 not only decreased phosphorylation of NF-κB induced by SuHx, but also reduced phosphorylation of the cytoplasmic inhibitor IκB (Figures 3E–H), which is a key event in activation of the NF-κB pathway (22). Next, mRNA expression of inflammatory cytokines of lungs in all the groups were measured by qRT-PCR. As shown in Figure 3I, ETRQβ-002 treatment effectively reduced mRNA expression of classical proinflammatory cytokines including IL-1β, IL-6, monocyte chemoattractant protein 1, and tumor necrosis factor α, some of which were significantly increased in patients with PAH (23, 24).

### ETRQβ-002 Attenuated Collagen Synthesis of PA and Inhibited Fibrosis Pathway in SuHx-Induced PAH

**Figure 4:**
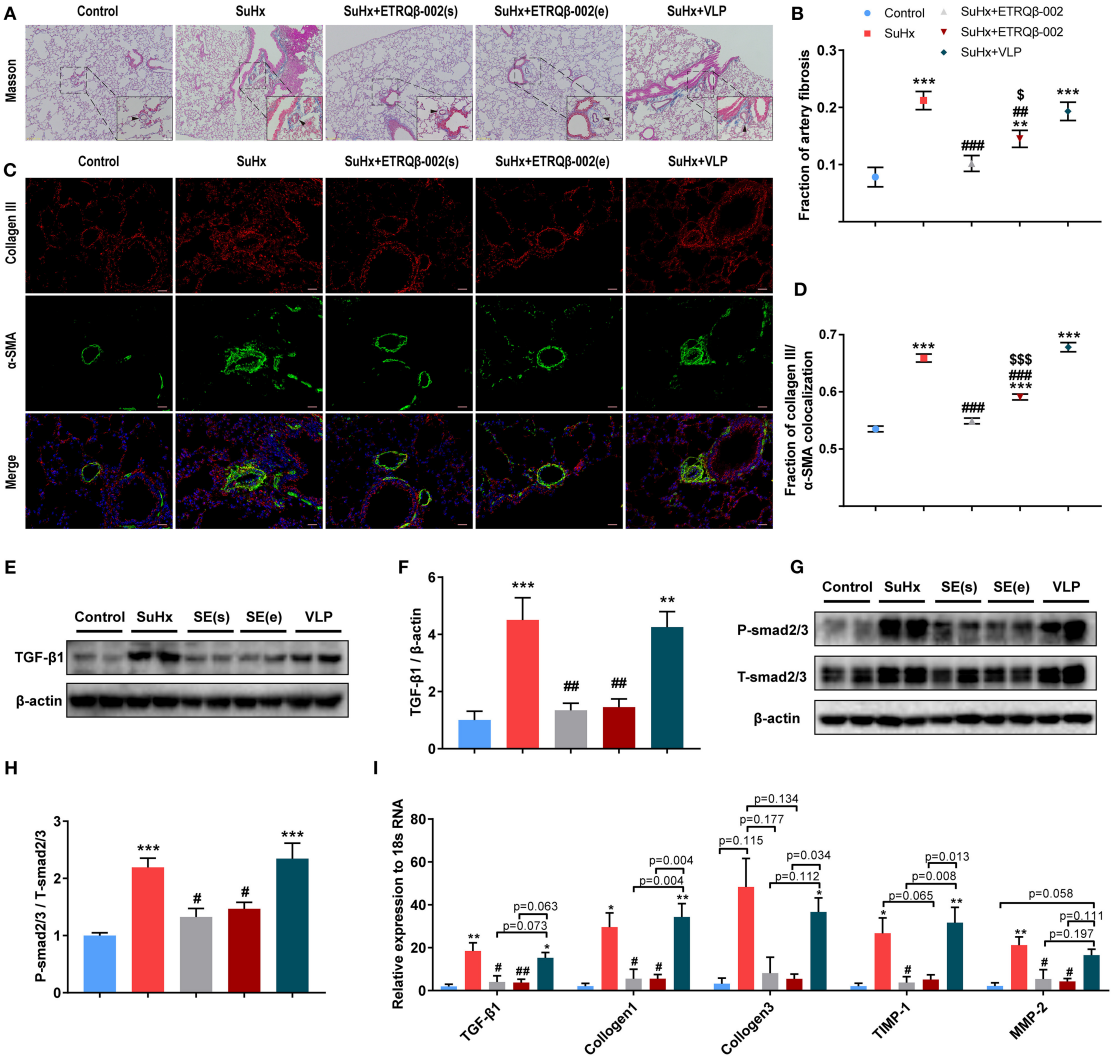
ETRQβ-002 vaccine effectively reduced fibrosis of PA in SuHx-induced PAH mice. **(A)** Representative photographs of Masson trichrome staining of lung sections from each group (scale bar = 50 μm). **(B)** Percentage of PA fibrosis burden (*n* = 6-8). **(C)** Representative photographs of collagen III/α-SMA double-immunofluorescence staining of lung sections from each group (scale bar = 20 μm). **(D)** The fraction of α-SMA–collagen III colocalization (*n* = 5). **(E,F)** Representative immunoblot, corresponding densitometric analysis of TGF-β1 (*n* = 6). **(G,H)** Representative immunoblot, corresponding densitometric analysis of psmad2/3 and total smad2/3 (n = 6). **(I)** Quantitative RT-PCR (qRT-PCR) analysis of fibrosis-related factors in lung homogenate from five groups (*n* = 4–5). The black arrows refer to the PAs. All data are expressed as the mean ± SEM. ^*^P < 0.05, ^**^P < 0.01, ^***^P < 0.001 vs. the control group. ^#^*P* < 0.05, ^*##*^*P* < 0.01, ^*###*^*P* < 0.001 vs. the SuHx group. ^*$*^*P* < 0.05, ^*$$$*^*P* < 0.001 vs. the SuHx + ETRQβ-002(s) group.

Aberrant accumulation of collagen and fibronectin in PA cause vessel stiffness, which is a crucial characteristic of vascular remodeling in PAH (25, 26). As shown in Figures 4A,B, Masson trichrome staining of the lung revealed that, compared to the control group, SuHx-induced PAH mice revealed a markedly higher percentage of collagen fibers in PA, which was significantly attenuated by ETRQβ-002 treatment. Further, immunofluorescence showed that ETRQβ-002 administration decreased the augmentation of α-SMA, collagen III, and α-SMA–collagen III colocalization in PA from SuHx-PAH mice (Figures 4C,D).

The TGF-β1/Smad2/Smad3 pathway plays an important role in cardiovascular fibrosis, and imbalance between TGF-β signaling and BMPRII signaling have been proven to contribute to PAH pathogenesis (27, 28). Western blotting showed that the expression of TGF-β1 and phosphorylation of smad2/3 in lung tissue homogenate were both decreased by ETRQβ-002 treatment (Figures 4E–H). Next, we examined mRNA expression of fibrosis-related factors in lung tissues in all the groups. Consistent with previous results, TGF-β1, collagen I, and collagen III were upregulated in SuHx-induced PAH mice, but all of those were downregulated in the ETRQβ-002 treatment groups (Figure 4I). Besides, ETRQβ-002 lowered mRNA expression of TIMP-1 and MMP-2 (Figure 4I), which are closely associated with extracellular matrix remodeling of PA and display increased production and activity in patients with IPAH and hypoxia-induced experimental PAH models (29). In general, these data support the conclusion that the ETRQβ-002 reduces collagen synthesis and fibrosis in PA through inhibiting the TGF-β1/Smad2/3 pathway.

### ETRQβ-002 Attenuated Right Ventricular Hypertrophy and Myocardial Fibrosis in SuHx-Induced PAH

**Figure 5:**
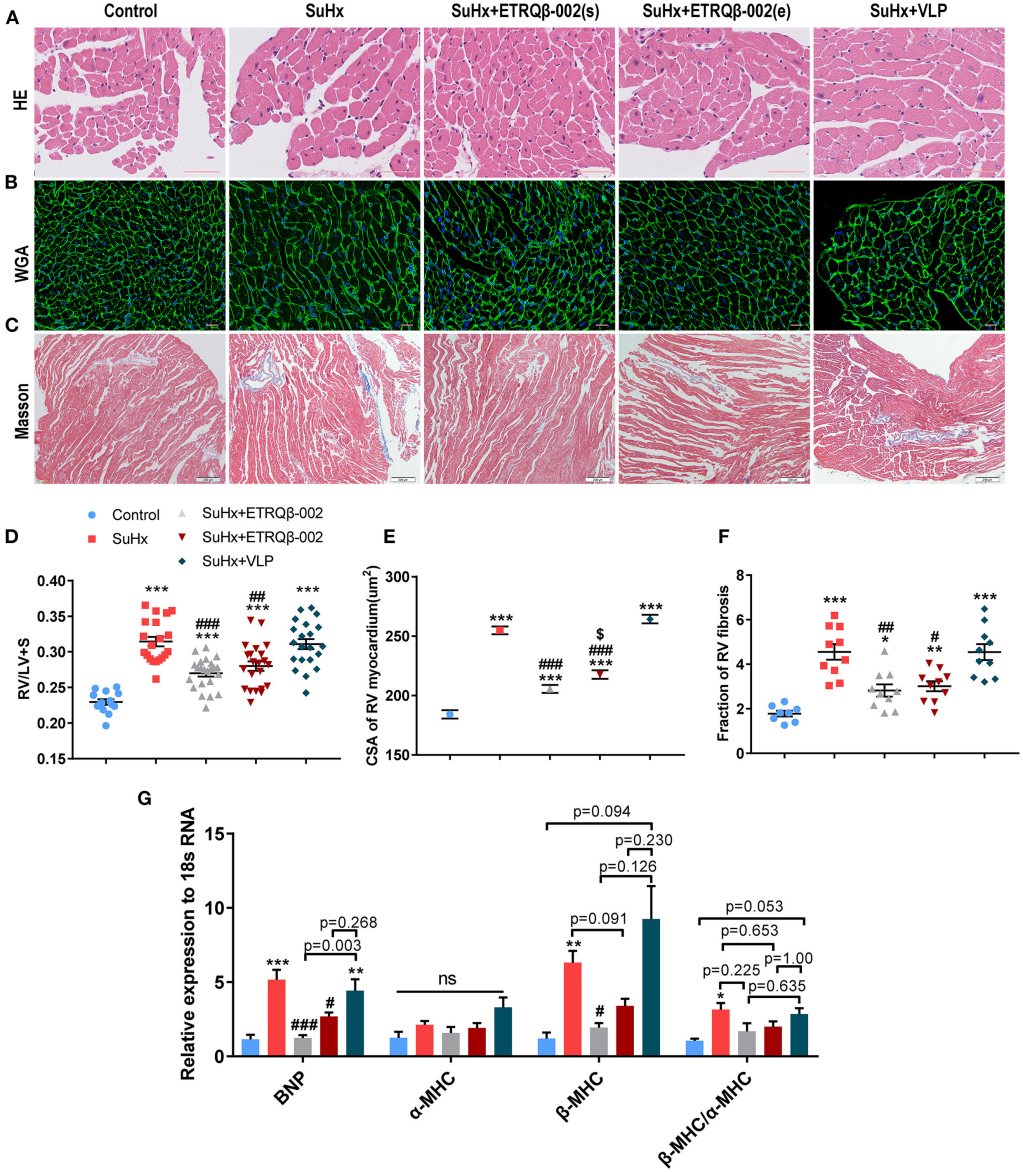
ETRQβ-002 vaccine provided a durable effect to attenuate right ventricular pathological remodeling in SuHx-induced PAH mice. **(A)** The representative photographs of hematoxylin-eosin (H&E) of the RV cardiomyocytes (scale bar = 50 μm). **(B)** The representative photographs of wheat germ agglutinin (WGA) of the RV cardiomyocytes (scale bar = 20 μm). **(C)** The representative photographs of Masson trichrome staining of the RV (scale bar = 200 μm). **(D)** The weight ratio of RV/(LV + IVS) (*n* = 14–23). **(E)** The cross sectional area (CSA) of RV cardiomyocytes (*n* = 6). **(F)** Percentage of RV myocardial fibrosis area (*n* = 8–10). **(G)** Quantitative RT-PCR (qRT-PCR) analysis of ventricular remodeling gene in RVs from five groups (*n* = 4–5). All data are expressed as the mean ± SEM. ^*^P < 0.05, ^**^P < 0.01, ^***^P < 0.001 vs. the control group. ^#^*P* < 0.05, ^*##*^*P* < 0.01, ^*###*^*P* < 0.001 vs. the SuHx group. ^*$*^*P* < 0.05 vs. the SuHx + ETRQβ-002(s) group. ns, no significant difference.

Longevity in patients with PAH is strongly determined by right ventricular function (30). Hence, RV compensatory remodeling, including cardiomyocyte hypertrophy and collagen synthesis, was investigated. As shown in Figures 5A,D, the weight ratio of RV/(LV + S) in the SuHx group was higher than the ETRQβ-002(s) group and ETRQβ-002(e) group (ETRQβ-002(s), 0.270 ± 0.005, *P* < 0.001; ETRQβ-002(e), 0.280 ± 0.007, *P* = 0.006; SuHx 0.314 ± 0.007). And, compared with those in the control group, cardiomyocytes in all groups treated with SuHx were obviously hypertrophied. The WGA staining revealed the CSA of RV myocardium in the ETRQβ-002(s) group and ETRQβ-002(e) group was significantly decreased compared to the SuHx group (ETRQβ-002(s), 205.5 ± 3.37 μm^2^, *P* < 0.001; ETRQβ-002(e), 217.6 ± 3.51 μm^2^, *P* < 0.001; SuHx 254.9 ± 3.31 μm^2^, Figures 5B,E). Besides, difference was observed between the ETRQβ-002(s) group and ETRQβ-002(e) group (ETRQβ-002(s) vs. ETRQβ-002(e), *P* = 0.013). Moreover, Masson trichrome staining of RV showed that ETRQβ-002 remarkably decreased the fibrosis induced by SuHx exposure (Figures 5C,F). In the SuHx group, the fibrotic area of RV was 4.55 ± 0.35%, whereas it was only 2.82 ± 0.27% in the ETRQβ-002(s) group (*P* = 0.009), and 3.01 ± 0.22% in the ETRQβ-002(e) group (*P* = 0.015).

Further, right ventricular gene expression affected by pressure overload was also assessed. qRT-PCR analysis showed that ETRQβ-002 effectively down-regulated the mRNA expression of brain natriuretic peptide (BNP) and β-MHC in RV tissues from SuHx-exposed mice, but the mRNA expression of α-MHC revealed no difference in all groups (Figure 5G). Compared with the SuHx group, the mRNA expression of ratio of β-MHC/α-MHC trended to be decreased in mice immunized with ETRQβ-002, but did not reach statistical significance (Figure 5G).

### No Safety Problems Were Observed in Vaccinated Mice

**Figure 6:**
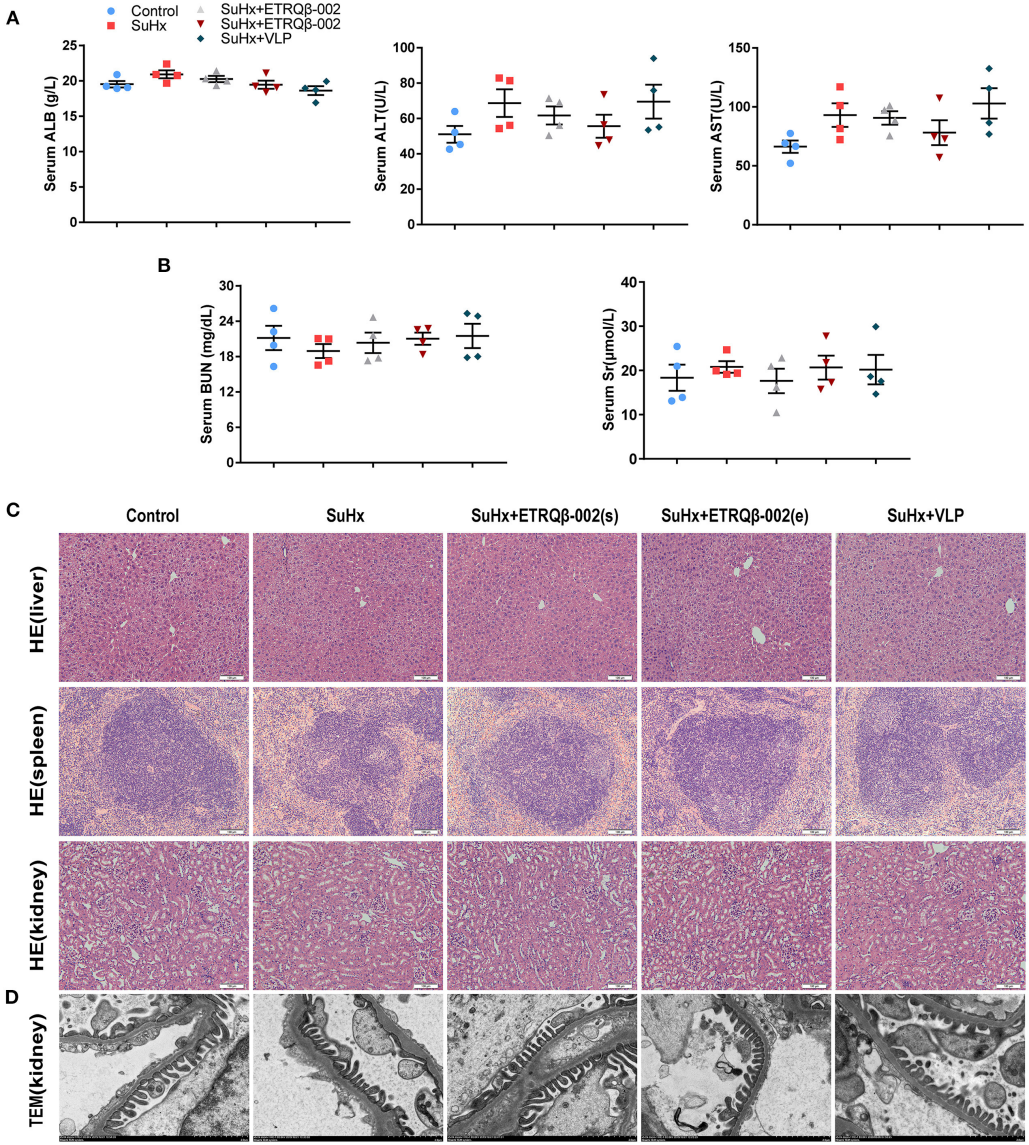
No immune-mediated injury was observed in mice injected with ETRQβ-002 vaccine during a long-term observation. **(A)** The hepatic function (ALT, AST, and ALB, *n* = 4). **(B)** The renal function [serum creatinine (Scr) and blood urea nitrogen (BUN), *n* = 4]. **(C)** The representative photographs of hematoxylin-eosin (HE) of the critical organs (liver, spleen, kidney) in the five groups (scale bar = 100 μm). **(D)** Transmission electron microscope image of kidney from the five groups (scale bar = 2 μm). All data are expressed as the mean ± SEM.

Safety endpoint of ETRQβ-002 is another concern. To evaluate long-term safety, we compared survival rates in all groups. As shown in Supplementary Figure 1, the survival rate had no obvious difference among groups except for the control group. Moreover, there were no significant differences in hemodynamic parameters including heart rate and systolic blood pressure measured by tail-cuff method in all groups (Supplementary Table 3). In addition, after multiple immunizations, biochemical analysis (including hepatic and renal function) did not reveal obvious abnormity in mice treated with ETRQβ-002 or VLP (Figures 6A,B). Moreover, histological results from important target organs (liver, spleen, and kidney) showed that no significant immune damage was detected in experimental mice (Figure 6C). TEM also showed no obvious injury or immune complex deposition in kidney of vaccinated mice (Figure 6D). These data are in accordance with our previous safety tests and further confirmed that ETRQβ-002 still displays a good safety during a long period of observation.

## Discussion

Current agents for PAH are required to be taken at least once or sometimes twice a day for many years, which appears to be a major challenge for patient compliance to their treatment. As a new approach for chronic diseases, therapeutic vaccine possesses some special advantages, including high specificity, durable effectiveness, and low cost (6). Previously, based on the structure and rationale of therapeutic vaccine, the peptide derived from the human ETAR was screened and covalently conjugated with VLP, which contains high repetitive epitopes presented on the surface and is effortless to bind to B cells through B cell receptor (BCRs). Then, self-antigen–carrier conjugate (ETRQβ-002 vaccine) could stimulate B cells to produce specific antibodies targeting ETAR. We demonstrated that ETRQβ-002 was able to moderate RV pressure elevation, PA remodeling, and RV hypertrophy in two types of PAH rodent models. Here, we confirmed the long-lasting protective effects of ETRQβ-002 in SuHx-induced PAH mice. Meanwhile, antiproliferation, anti-inflammatory, and antifibrosis effects in PAs were also observed in PAH mice vaccinated with ETRQβ-002.

In order to investigate the long-term therapeutic effects of ETRQβ-002 during the establishment of PH, mice were administered SuHx and vaccine simultaneously in the SuHx + ETRQβ-002(s) group. Meanwhile, to examine the effects of the vaccine in well-established PH, mice in the SuHx + ETRQβ-002(e) group were vaccinated after SuHx treatment finished. Results showed that the ETRQβ-002 induced high-level antibody titers, significantly reduced RVSP by about 7 mm Hg, and improved pathological remodeling of PAs in SuHx mice.

It is well-described that continuous proliferation of pulmonary arterial smooth muscle cells is the crucial pathological characteristics of PAH, leading to medial hypertrophy and progressive occlusion of distal pulmonary resistant vessels (14). ET-1 initiates a series of signal cascades, such as MAPK, Akt, and β-catenin, and activated MAPK signaling which exhibits a strong proliferative effect in cancer has also been implicated in PAH (14, 18, 31). Our previous data demonstrated that anti–ETR-002 antibody inhibited ET-1–induced ERK1/2 phosphorylation *in vitro*. Here, we further confirmed the antiproliferation effect of ETRQβ-002 *in vivo*. Histological and biochemical analyses showed that ETRQβ-002 effectively reduced PA thickening and numbers of proliferating marker cells in vessels and perivascular tissues. Phosphorylation of ERK1/2 and p38, as well as PAH-related growth factors, was also down-regulated in lung of SuHx-induced PAH mice vaccinated with ETRQβ-002. These results suggested that ETRQβ-002 displayed a considerable antiproliferation potency in experimental PAH, similar to the effect of endothelin-receptor antagonists in preclinical experimental cancer models (12).

Recent researches have shown the critical role of inflammation in pulmonary vascular remodeling (23). Infiltration of inflammatory cells and proinflammatory cytokines cause excessive proliferation of pulmonary artery endothelial cells and smooth muscle cells. In PAH patients, it has been observed that the numbers of inflammatory cells and levels of proinflammatory cytokines are elevated (23, 24). NF-κB is a major regulator in immunity and inflammatory responses, which could be activated by ET-1 in arterial myocytes (32). Recently, the critical roles of NF-κB in the pathophysiology of PAH have been reported, and inhibition of NF-κB alleviated inflammation in MCT-PAH animals (23, 33). Our data indicated that ETRQβ-002 significantly reduced lymphocytes cell infiltration in pulmonary artery and its periphery. Additionally, activity of NF-κB signal path, as well as the mRNA expression of inflammatory factors in SuHx-induced PAH, was also reduced. These results implied that ETRQβ-002 is able to suppress the inflammatory response and improve PA remodeling in experimental PAH.

Increased depositions of collagen and elastin are known to be important determinants of medial thickening during the progression of PAH, and pulmonary vascular remodeling is characterized, somewhat, by a fibrotic vasculopathy in small PAs in PAH (3, 34). A few studies have revealed that stiffening and decreased compliance in pulmonary vasculature occur in the early stage of PAH, even before the increase of pulmonary arterial pressure (35, 36), and thus could better reflect the prognosis of PAH than PVR (37, 38). ET-1 is fundamental to the pathogenesis of fibrosis and may be a pivotal intermediary of profibrotic effects of other mediators. Endothelin-receptor antagonists have been applied to various fibrosis-related diseases including idiopathic pulmonary fibrosis and chronic kidney disease (12); hence, we speculated ETRQβ-002 may also have an antifibrosis effect. Here, we found that ETRQβ-002 did ameliorate collagen deposition in PA, blocked classical fibrosis signaling pathway, as well as reduced the mRNA expression of fibrosis-related factors in SuHx-induced PAH.

The prognosis of patients with PH is largely determined by the structure and function of RV. Initially, the RV adapts to the increased pressure overload by compensatory processes, including cardiomyocyte hypertrophy, elevated capillary density, and synthesis of extracellular matrix proteins. Finally, as contractile weakening progresses, decompensated right ventricular failure characterized by contractile dysfunction, extensive fibrosis, chamber dilatation, and capillary rarefaction occurs, leading eventually to death (17, 30). In our study, significant RV pathological remodeling, including hypertrophy and fibrosis, and up-regulated mRNA expression of ventricular wall stress proteins BNP and adult and fetal isoforms of myosin heavy chain β-MHC were found in SuHx-induced PAH mice. ETRQβ-002 significantly attenuated these changes. Besides, we found that the therapeutic efficacy of ETRQβ-002 in experimental PAH might be associated with the time of administration, because data showed that protective effects of the vaccine in the SuHx + ETRQβ-002(s) group were better than that in the SuHx + ETRQβ-002(e) group in some aspects, including the proliferation of PA (Ki67), fibrosis of PA, and CSA of RV. These results suggest that earlier vaccination with ETRQβ-002 might provide better protection.

The long-term safety examination of the vaccine has also been conducted in this study. Although the vaccine did not significantly improve the long-term survival rate of SuHx-PAH mice, the obvious side effect was not observed. As the results revealed, multiple ETRQβ-002 immunizations caused no injury in hepatic and renal function and did not lead to immune damage in important organs of mice. Further, TEM showed that no immune-mediated damage was observed in the kidney of mice immunized with ETRQβ-002. In principle, the safety of the ETRQβ-002 vaccine mainly includes the safety of the ETR-002 peptide and Qβ VLP carrier separately. First, as the ETR-002 peptide is only 10 amino acids in length, which alone does not include a T-cell epitope, it is not able to induce a T-cell response. Thus, Tc will not be activated and does not produce an overall antiendothelial response, just a specific anti-ETAR from B cells. Second, our previous work found that the peptide–VLP vaccine strongly activated dendritic cells, which promoted T-cell differentiation to follicular helper T cells instead of Tc or inflammation-related T_H_1/T_H_2/T_H_17, which showed the good safety of the peptide–VLP vaccine (39). Moreover, several preclinical and clinical trials have shown a good safety of peptide–VLP vaccines (40–42). All of these pieces of evidence indicate a long-term safety endpoint of ETRQβ-002.

Our study has some limitations. First, we used only one model (SuHx mice model), which has a few disadvantages including vascular remodeling and partial recovery of RVSP after exposure to normoxia. However, the RVSP of SuHx mice (hypoxia combined SU5416 injection) will not fully recover even after a long-term normoxic recovery compared with the VehHx mice (hypoxia alone) (43). Moreover, the results in this study showed that ETRQβ-002 decreased the RVSP (approximately 7 mm Hg) and improved the remodeling of PAs compared with the SuHx group; thus, the long-term effects of the ETRQβ-002 could be confirmed. Second, although the safety of the ETRQβ-002 has been initially verified, a more precise long-term safety endpoint of the vaccine needs to be further investigated.

## Conclusion

In summary, our study demonstrated that ETRQβ-002 offers a long-term efficacy in reducing RV pressure and RV remodeling. The vaccine also attenuates crucial pathological process in PAs in SuHx-induced PAH, including proliferation, inflammation, and fibrosis.

## Data Availability Statement

The original contributions presented in the study are included in the article/Supplementary Material, further inquiries can be directed to the corresponding author/s.

## Ethics Statement

The animal study was reviewed and approved by Ethics Committee of Tongji Medical College of Huazhong University of Science and Technology, China.

## Author Contributions

YD, ZQ, and WM designed the study. YD and ML wrote the paper. CL, XC, and XS performed the research. ZB, DS, JZ, and GP analyzed data. YL, ZQ, and ZZ supervised the research and obtained resources and fundings. All authors contributed to the article and approved the submitted version.

## Conflict of Interest

The authors declare that the research was conducted in the absence of any commercial or financial relationships that could be construed as a potential conflict of interest.
