# Supplementary material for: Long-Term Effect of a Vaccine Targeting Endothelin-1 Receptor Type A in Pulmonary Arterial Hypertension
Source: Front Cardiovasc Med. 2021 Jun 17;8:683436. doi: 10.3389/fcvm.2021.683436 (PMC8247646; doi:10.3389/fcvm.2021.683436)
Supplement: Supplementary file 1 [file Table_1.DOC]

**Supplementary Material**

**Supplement Tables**

**Table S1. Primers sequence for quantitative real-time PCR**

| Gene |  | Sequence(5'-3') |
| --- | --- | --- |
| PDGF-α | forward | CTCTGCCATCATACCTTGCC |
|  | reverse | AAACATTAAACTCGCTGGTCTTG |
| PDGF-β | forward | GCCTTAGTGGTCCTTACCGTCAT |
|  | reverse | GAGTGCGTCCCAGAACAAGC |
| FGF | forward | GCGACCCACACGTCAAACTA |
|  | reverse | TCCCTTGATAGACACAACTCCTC |
| VEGF | forward | GCACATAGAGAGAATGAGCTTCC |
|  | reverse | CTCCGCTCTGAACAAGGCT |
| G-CSF | forward | GCAGGCTCTATCGGGTATTTCC |
|  | reverse | GCAACATCCAGCTGAAGCAA |
| M-CSF | forward | TGATTGGGAATGGACACCTG |
|  | reverse | AAAGGCAATCTGGCATGAAGT |
| TGF-β | forward | GATACGCCTGAGTGGCTGTCTT |
|  | reverse | GGGGCTGATCCCGTTGATT |
| Collagen-Ⅰ | forward | AAGAAGCACGTCTGGTTTGGAG |
|  | reverse | GGTCCATGTAGGCTAAGATGTT |
| Collagen-Ⅲ | forward | CCTGGTGCTATTGGTCCATCT |
|  | reverse | CGTCCATCAAAGCCTCTGTGT |
| TIMP-1 | forward | GCAACTCGGACCTGGTCATAA |
|  | reverse | CGGCCCGTGATGAGAAACT |
| MMP-2 | forward | CAAGTTCCCCGGCGATGTC |
|  | reverse | TTCTGGTCAAGGTCACCTGTC |
| IL-1β | forward | AGAAGATGGAAAAACGGTT |
|  | reverse | CTTGTGCTCTGCTTGTGAG |
| IL-6 | forward | GAAAATCTGCTCTGGTCTTCT |
|  | reverse | CACTCCTTCTGTGACTCTAAC |
| MCP-1 | forward | TTGAGGACAGACACAGCAGCC |
|  | reverse | TCACCAATTCCCCTAGCACC |
| TNF-α | forward | ATGTCTCAGCCTCTTCTCATTC |
|  | reverse | GCTTGTCACTCGAATTTTGAGA |
| BNP | forward | CTGCTTTTCCTTAATCTGTCG |
|  | reverse | AGAAGTTCTTTTGTAGGGCCT |
| α-MHC | forward | GCCCAGTACCTCCGAAAGTC |
|  | reverse | GCCTTAACATACTCCTCCTTGTC |
| β-MHC | forward | ACTGTCAACACTAAGAGGGTCA |
|  | reverse | TTGGATGATTTGATCTTCCAGGG |
| 18sRNA | forward | GAATTCCCAGTAAGTGCGGG |
|  | reverse | GGGCAGGGACTTAATCAACG |

**Table S2. Antibodies information in Western blotting**

| Product | Item No | Dilution ratio | Company |
| --- | --- | --- | --- |
| Phospho-Erk1/2 | no. 4370 | 1:1000 | Cell Signaling Technology |
| Erk1/2 | no. 4696 | 1:1000 | Cell Signaling Technology |
| Phospho-p38 | no. 9211 | 1:1000 | Cell Signaling Technology |
| Total-p38 | no. 9212 | 1:1000 | Cell Signaling Technology |
| Phospho-p65 | no. 3039 | 1:1000 | Cell Signaling Technology |
| Phospho-IκBα | no. 2859 | 1:1000 | Cell Signaling Technology |
| Total- IκBα | no. 4814 | 1:1000 | Cell Signaling Technology |
| Phospho-smad2/3 | no. 8828 | 1:1000 | Cell Signaling Technology |
| Total- smad2/3 | no. 8685 | 1:1000 | Cell Signaling Technology |
| TGF-β1 | 21898-1-AP | 1:1000 | Proteintech |
| β-actin | 60008-1-lg | 1:1000 | Proteintech |

**Table S3.** Parameter of SuHx-PAH mice

| **Day** | **Parameter** | **Control** | **SuHx** | **SuHx + ETRQβ-002(s)** | **SuHx +ETRQβ-002(e)** | **SuHx +VLP** |
| --- | --- | --- | --- | --- | --- | --- |
| 0 | SBP(mmHg) | 128±3 | 130±3 | 127±3 | 124±3 | 123±2 |
|  | DBP(mmHg) | 76±2 | 78±2 | 75±2 | 76±3 | 78±3 |
|  | HR(bpm) | 564±7 | 553±8 | 549±8 | 577±6 | 574±5 |
| 56 | SBP(mmHg) | 123±2 | 123±3 | 124±3 | 127±2 | 122±2 |
|  | DBP(mmHg) | 72±2 | 72±1 | 74±2 | 77±2 | 76±1 |
|  | HR(bpm) | 538±8 | 539±9 | 559±11 | 543±9 | 554±14 |
| 112 | SBP(mmHg) | 126±2 | 122±3 | 125±1 | 128±3 | 121±3 |
|  | DBP(mmHg) | 72±3 | 70±2 | 77±2 | 75±1 | 71±1 |
|  | HR(bpm) | 558±13 | 543±7 | 558±12 | 562±11 | 554±13 |

DBP = diastolic blood pressure; HR = heart rate; SBP = systolic blood pressure.

**Figure S1.** Survival rate of SuHx-PAH mice
